# Supplementary material for: Asynchronous magnetic resonance elastography: Shear wave speed reconstruction using noise correlation of incoherent waves
Source: Magn Reson Med. 2022 Oct 27;89(3):990–1001. doi: 10.1002/mrm.29502 (PMC9792433; doi:10.1002/mrm.29502)
Supplement: Supplementary file 1 — DATA S1 MATLAB code used to generate the numerical simulations of Figure 1 [file MRM-89-990-s002.zip › k-Wave/helpfiles/envelopeDetection.html]

envelopeDetection :: Functions (k-Wave)


# envelopeDetection

Extract signal envelope using the Hilbert Transform.

## Syntax

```
env = envelopeDetection(x)
```

## Description

`envelopeDetection` applies the Hilbert transform to extract the envelope from an input vector `x`. If `x` is a matrix, the envelope along each row is returned. For example, running the code

```
% create tone burst
x = toneBurst(10e6, 0.5e6, 10);

% plot tone burst and envelope
figure;
plot(0:length(x) - 1, x, 'k-', 0:length(x) - 1, envelopeDetection(x), 'r-');
legend('Input Signal', 'Envelope');
```

produces the output

## Inputs

|  |  |
| --- | --- |
| `x` | input function |

## Outputs

|  |  |
| --- | --- |
| `env` | envelope of input function |

## See Also

`fft`
